# Clinical translation of liquid biopsy in early cancer screening: biofluid selection, model construction, and clinical validation

**DOI:** 10.3389/fonc.2026.1946352

**Published:** 2026-10-26

**Authors:** Pin Cui, Shu Xu, Xiaohui Lin, Shiyi Song, Bingding Huang, Zhiming Xu

**Affiliations:** 1 Shenzhen Rapha Biotechnology Inc., Shenzhen, Guangdong, China; 2 Cancer Center, Shenzhen University of Advanced Technology General Hospital, Shenzhen, Guangdong, China; 3 Department of Oncology, Shenzhen Key Laboratory of Gastrointestinal, Cancer Translational Research, Cancer Institute, Peking University Shenzhen Hospital, Shenzhen, China; 4 Department of Endoscopy, The First Affiliated Hospital of Henan Medical University, and Henan Key Laboratory of the Prevention and Treatment of Gastrointestinal Cancer, Weihui, Henan, China; 5 School of Artificial Intelligence, Shenzhen Technology University, Shenzhen, Guangdong, China

**Keywords:** biomarkers, clinical validation, early cancer detection, liquid biopsy, multi-omics

## Abstract

Global cancer incidence continues to rise, underscoring the urgent need for effective early detection strategies. Liquid biopsy shows promise as a minimally invasive approach, yet only a limited number of assays have successfully transitioned from research to clinical practice. This review examines the critical decisions and challenges encountered along the translational pathway for liquid biopsy based early cancer screening, covering biofluid selection, biomarker characteristics, multi-omics integration, model construction, and the hierarchy of clinical evidence. We highlight the importance of biologically driven complementarity in biomarker combination, rigorous control group design, and prospective real-world validation, while also identifying key gaps that currently constrain clinical adoption. Collectively, these considerations aim to inform more efficient development strategies and help bridge the gap between technological innovation and clinically impactful tests.

## 1 Introduction

Cancer poses a global health burden as the second leading cause of death worldwide, accounting for nearly 10 million deaths annually (1) and representing almost one in every six deaths (2). Approximately 19.3 million new cancer cases are reported each year, and this number is projected to exceed 35 million by 2050, highlighting the immense scale of the disease (3, 4). In this context, early diagnosis and intervention are critical for improving outcomes and reducing healthcare costs (5–7), given that metastatic lesions account for roughly 90% of cancer-related deaths and five-year survival rates decline steadily with advancing stage (8, 9). This stage-dependent decline in survival also carries severe economic results, as treating advanced-stage cancers incurs dramatically higher costs compared to early-stage disease due to extended hospital stays and increased interventions (10, 11).

Early diagnosis can improve surgical outcomes and reduce costs, but existing clinical tests lack the sensitivity and specificity needed to achieve these goals reliably. Standardized screening protocols exist only for a few cancer types and cover a limited portion of the population, detecting annually just 24% of cancer diagnoses in the UK, 28% in the US, and 63% in China (12). Screening methods such as low-dose computed tomography (LDCT) for lung cancer (13) and the Pap smear for cervical cancer frequently face adherence challenges (14). The high false-positive rate of computed tomography can trigger invasive follow-ups and cause patient anxiety (2, 12), while the physical discomfort and embarrassment of Pap smears present a major barrier to uptake (14). These limitations result in suboptimal signal-to-noise ratios, and the coordination of multiple tests further increases healthcare costs and decreases operational efficiency.

To overcome these diagnostic bottlenecks, liquid biopsy has emerged as a promising alternative. Unlike traditional tissue biopsy which is invasive and fundamentally unsuitable for asymptomatic screening because it relies on prior imaging to locate a tumor, liquid biopsy offers a proactive solution. By utilizing a diverse range of bodily fluids (e.g., blood, urine, saliva), liquid biopsy provides a flexible and repeatable approach for cancer detection (4, 15). This method captures a comprehensive molecular profile of the disease overcoming the spatial limitations and sampling bias of localized tissue biopsies. These liquid samples contain a rich repository of circulating biomarkers derived from tumors, such as cell-free DNA (cfDNA), cell-free RNA (cfRNA), proteins, circulating tumor cells (CTCs), and extracellular vesicles (EVs) (16). Applying these circulating biomarkers to early detection remains challenging because early-stage tumors release exceptionally weak molecular signals that are heavily diluted by normal physiological noise. To overcome this limitation, researchers must carefully evaluate various analytes to effectively amplify the tumor signal (17). For example, analyzing epigenetic changes in cfDNA such as methylation or fragmentation patterns can provide a highly sensitive and specific avenue for capturing early and weak tumor signals (18, 19).

Although liquid biopsy research has grown rapidly and accumulated many promising studies, only a few early screening products have successfully made it to actual clinical use. In this review, we explore the translational pathway from biofluid selection to clinical implementation, covering biofluid choice, biomarker integration, model building, and clinical validation as four interconnected stages (**Figure 1**), each with its own decisions and pitfalls. To ground our discussion in real data, we compiled about 80 single-cancer and 20 pan-cancer studies, providing a landscape of biofluid types, biomarkers, control group selections, clinical trial phases, and validation cohort sizes across cancer types. Beyond descriptive summaries, we analyze key methodological issues, including how evidence levels attenuate across trial phases and why higher-grade trials are essential for validation. We also discuss practical barriers such as assay standardization, regulatory pathways, reimbursement, and clinical workflow integration, which are often overlooked but critically affect patient access. Rather than cataloging emerging technologies, this review focuses on core principles and persistent gaps that we hope may inform more efficient development strategies.

**Figure 1:**
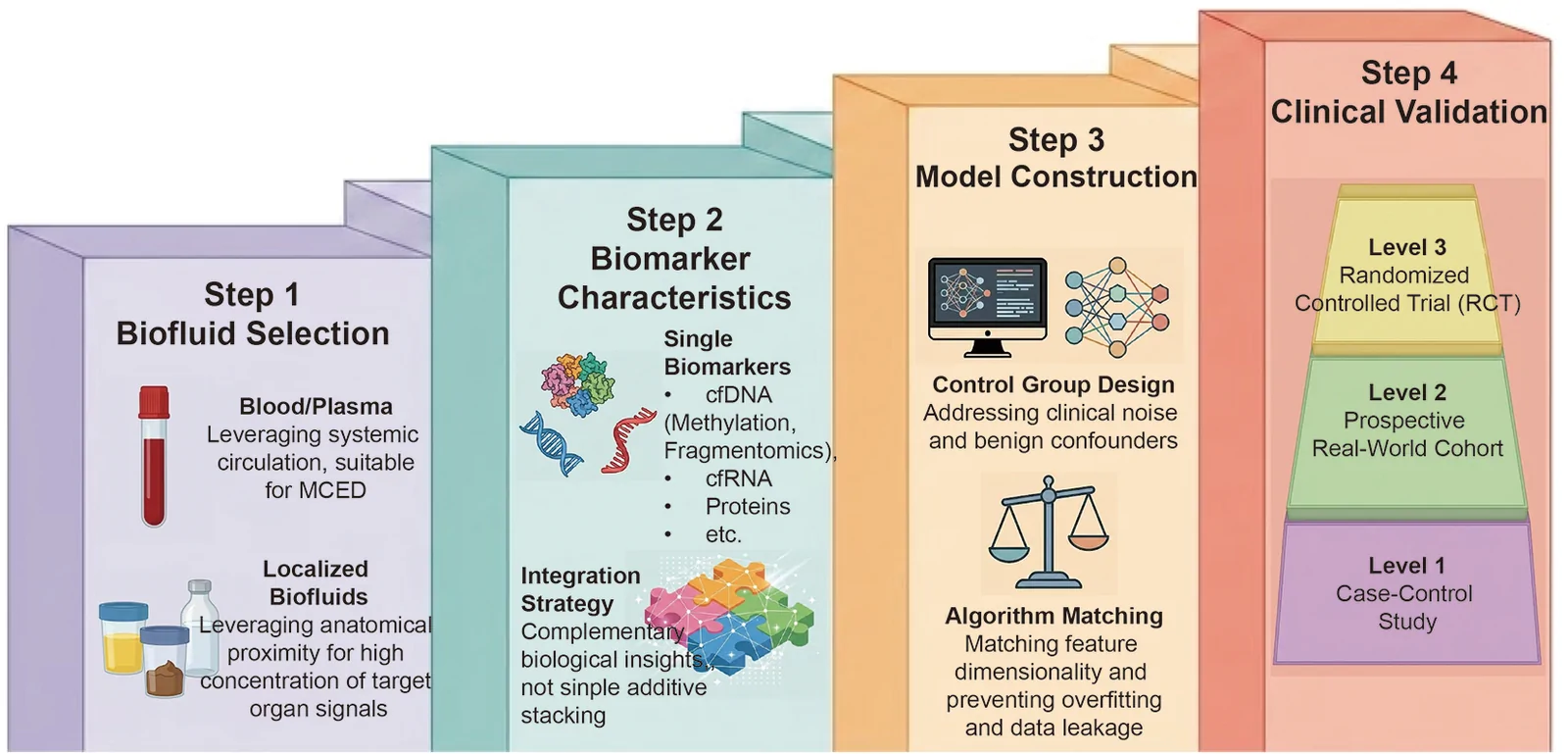
Translational framework for liquid biopsy in early cancer detection. This schematic outlines the four critical stages in the clinical translation of liquid biopsy-based early cancer screening assays. Step 1: Biofluid Selection: The choice depends on the clinical goal, with systemic circulation (plasma) enabling MCED and localized biofluids leveraging anatomical proximity to enrich target organ signals. Step 2: Biomarker Characteristics: This phase involves selecting single biomarkers (such as cfDNA methylation, fragmentomics, and etc.) and employing an integration strategy driven by biological complementarity rather than simple additive stacking. Step 3: Model Construction: Model reliability requires rigorous control group design to address clinical noise and benign confounders; alongside careful algorithm matching to align with feature dimensionality and prevent overfitting and data leakage. Step 4: Clinical Validation: Evidence generation follows a hierarchical ladder progressing from Level 1 Case-Control studies to Level 2 Prospective Real-World Cohorts, and ultimately to Level 3 RCTs to confirm clinical benefit.

## 2 Search strategy and selection criteria

To survey the current landscape of early cancer detection research, we conducted a targeted literature search in PubMed using keywords including “liquid biopsy,” “early cancer detection,” combined with cancer-specific terms for eight major malignancies: lung cancer (LC), colorectal cancer (CRC), hepatocellular carcinoma (HCC), gastric cancer (GC), breast cancer (BC), prostate cancer (PCa), bladder cancer (BCa), and cervical cancer (CC). The search was restricted to studies published between January 2020 and August 2026 that reported performance metrics for early-stage cancer detection. We reviewed English language papers only. Following the initial retrieval, we manually screened the titles and abstracts of all records to identify studies specifically addressing early detection applications, without imposing additional exclusion criteria beyond relevance to the topic. Through this process, we compiled about 10 studies per cancer type. For the pan-cancer studies, rather than applying the same systematic search strategy, we selected 20 key publications based on our understanding of the pan-cancer early detection research landscape. In this selection, we prioritized studies that exemplified distinct technological approaches (e.g., fragmentomics as in the DELFI assay, protein-based markers as in CancerSEEK) or represented large-scale clinical trials. Additionally, when the same commercial entity (e.g., GRAIL) had published both case-control and prospective studies using the same underlying technology, we included both to illustrate the performance trajectory across evidence levels. These single-cancer and pan-cancer studies are summarized in **Figure 2** (20–104) and **Figure 3** (18, 19, 105–121), respectively.

**Figure 2:**
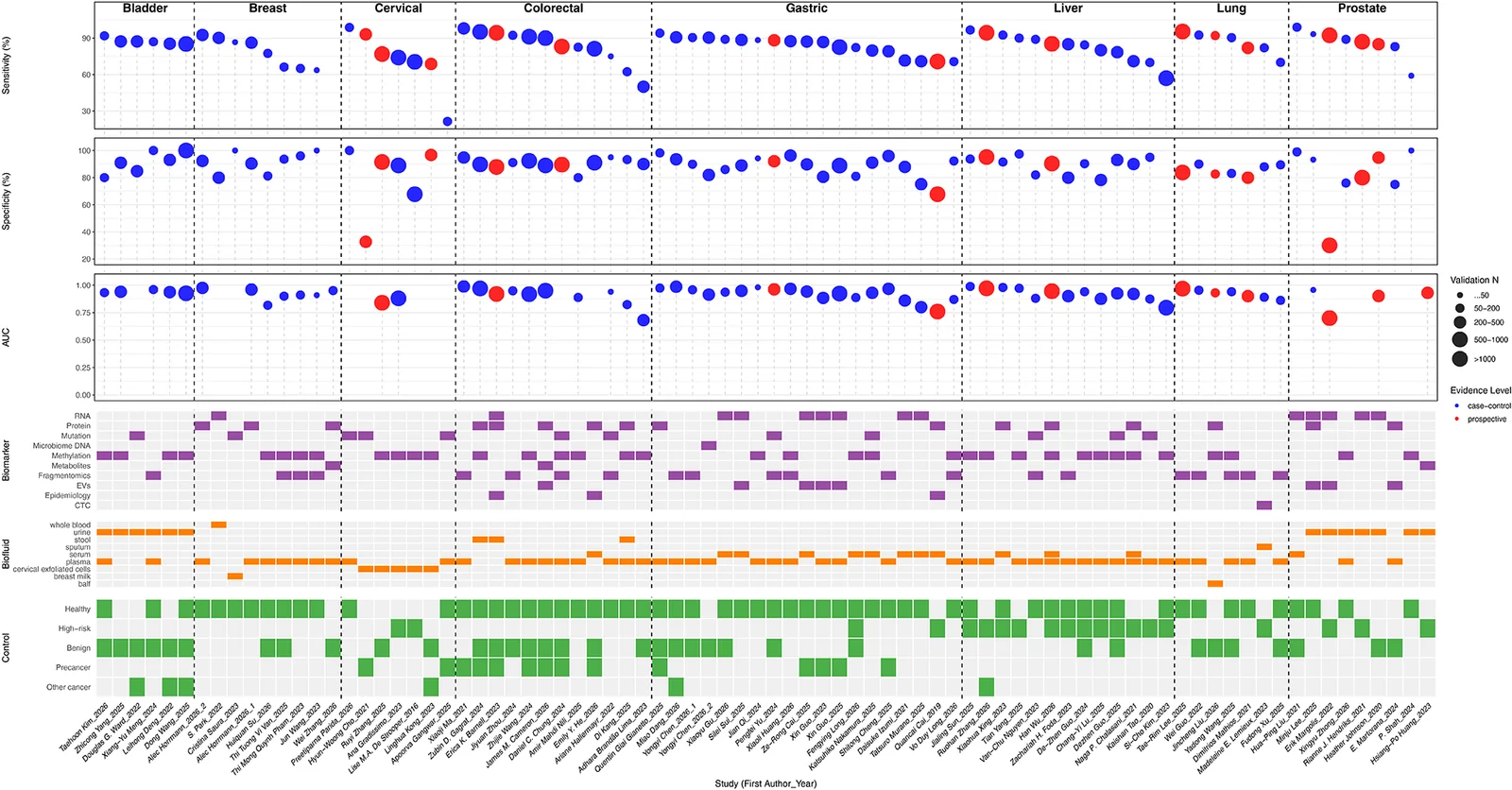
Summary of liquid biopsy studies for early detection of eight cancer types. The image consists of six stacked panels sharing the same x-axis order. Studies are grouped by cancer type and sorted by descending sensitivity within each type. Dashed vertical lines separate cancer types. The top panel shows sensitivity for each study, with points colored by evidence level (e.g., prospective or case-control) and sized by validation cohort size groups (≤50, 50-200, 200-500, 500-1000, >1000). The second panel shows specificity, and the third shows AUC. The fourth panel is a heatmap of biomarker categories used (purple tiles indicate use, gray tiles indicate not used). The fifth panel shows biofluid categories (orange tiles). The sixth panel shows control group categories (green tiles), including Healthy, High-risk, Benign, Precancer, and Other cancer. The x-axis of the bottom panel displays study identifiers (first author and year). For studies using multiple fluids, biomarkers, or controls, each category is marked separately.

**Figure 3:**
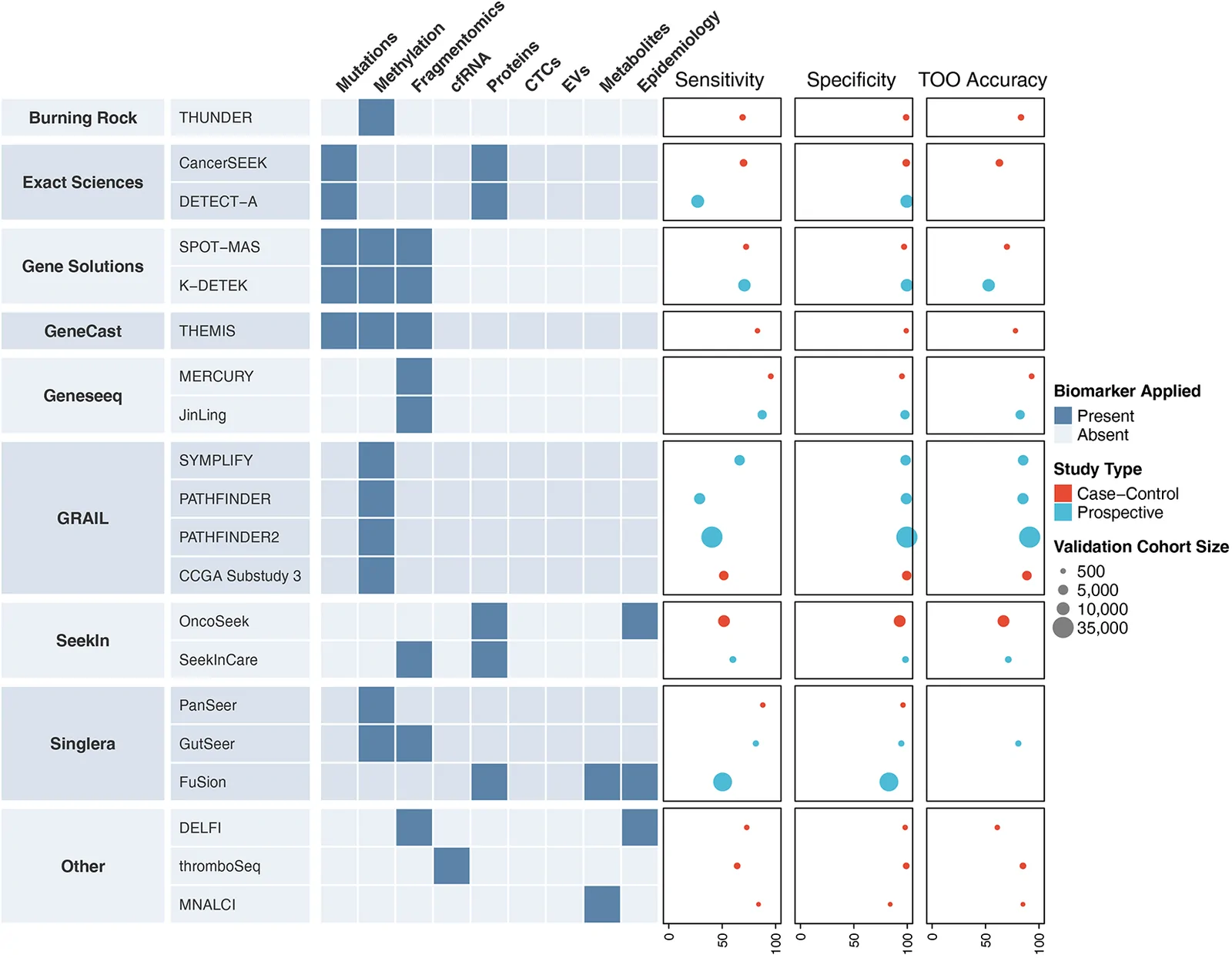
Summary of MCED studies. The image is organized into three sections from left to right. The left section shows company names with alternating background shading, and studies are grouped by company. The middle section contains a heatmap indicating the use of seven biomarker categories (Mutations, Methylation, Fragmentomics, cfRNA, Proteins, CTCs, EVs, Metabolites and Epidemiology). Blue tiles indicate that a biomarker category was used, and gray tiles indicate that it was not used. The right section presents three scatter plots for each study, showing Sensitivity (%), Specificity (%), and TOO (Tissue of Origin) accuracy (%), all with a y-axis from 0% to 100%. In these scatter plots, all points are circles. The color of each circle denotes the study type (Case Control in red or Prospective in blue), and the size of each circle reflects the validation cohort size (larger circles indicate larger cohorts). Missing values are not plotted because they would fall outside the visible y-axis range. Detailed legends for biomarker use, study type, and validation cohort size are provided alongside the image.

## 3 Biofluid selection — different samples for liquid biopsy

Liquid biopsy samples are broadly divided into blood and local body fluids. Blood, as the most commonly used sample source, reaches virtually every tissue via the circulatory system, allowing it to collect tumor-shed materials regardless of anatomical location and serving as a common reservoir for tumor-specific signals (122). This feature makes blood an ideal medium for multi-cancer early detection (MCED). For certain anatomical sites, local body fluids can enrich higher concentrations of tumor signals due to their proximity, sometimes outperforming plasma in detection sensitivity (15, 123, 124). These local fluids include urine, stool, cerebrospinal fluid, sputum, bronchoalveolar lavage fluid (BALF), cervical exfoliated cells, and saliva, among others (4, 123). Tumor-derived molecular biomarkers are primarily generated through mechanisms such as apoptosis, necrosis, active secretion, or mechanical shedding (125, 126). When encountering specific anatomical barriers, such as the blood-brain barrier (127), the glomerular filtration barrier (128), or the alveolar-capillary barrier (129), these substances are retained and become specifically distributed in the corresponding body fluid compartments. Consequently, depending on their anatomical location and physiological characteristics, various local body fluids capture tumor-derived biomarkers, including cfDNA, proteins, cfRNA, and exosomes, in different ways.

Comprising plasma and serum, blood is the preferred and most widely used sample type, offering significant advantages such as non-invasive repeatable collection, superior ctDNA enrichment, lower genomic DNA contamination, and higher stability than serum (130). Nonetheless, plasma-based detection faces physiological challenges. The total plasma volume in the human body is approximately 4–5 L, and the various tumor molecular biomarkers are massively diluted upon entry, often reaching only trace levels (123, 131). Moreover, their short half-lives, such as ctDNA’s half-life of about 16 minutes to 2.5 hours, render the signals highly labile (132). At the same time, background noise from non-tumor-derived mutations, such as clonal hematopoiesis, further complicates accurate identification (133). Once tumor signals enter the blood, their abundance varies dramatically across different stages and cancer types. Taking ctDNA as an example, one study reported that the peripheral blood ctDNA detection rate in early-stage (stage I) patients is significantly lower than that in late-stage (stage IV) patients (47% vs. 82%), and that the concentration of mutant fragments in plasma rises stepwise with advancing stage (134). Across cancer types, tumors arising in highly vascularized organs such as the liver, lung, stomach, and colorectum shed signals more readily into the circulation, whereas primary brain tumors are hindered by the blood-brain barrier, and thyroid, renal, and prostate cancers tend to release lower concentrations of tumor molecules into plasma owing to their intrinsic biological features (134). These variations stem from multifactorial interactions, including tumor size and activity, local physical barriers, vascular supply, and systemic circulatory and metabolic conditions.

Owing to anatomical proximity, many tumors can directly release signals into surrounding fluids, leading to much higher concentrations of tumor biomarkers in local body fluids than in plasma (123). A classic example is the detection of KRAS mutations in paired urine and plasma samples from patients with early-stage colorectal cancer, where the sensitivity of trans-renal tumor DNA (trtDNA) in urine reached 37.7%, significantly outperforming plasma ctDNA, which achieved only 21.3% (*P* = 0.047) (135). Similarly, in central nervous system lymphomas, ctDNA detection rates in cerebrospinal fluid reached 100%, markedly superior to the 78% detection rate in plasma (136). These findings indicate that anatomically proximal fluids can provide stronger tumor-derived signals than peripheral blood, depending on the clinical context. Local body fluids can be categorized according to their collection method into non-invasive and invasive types. Non-invasively collected samples, such as urine, stool, sputum, cervical exfoliated cells, and saliva used for oral and head/neck cancers, are similar to plasma in that they can be obtained repeatedly and are well suited for large-scale screening of asymptomatic populations (15). Invasively collected samples, such as bile, BALF, and pleural or ascitic fluid, are typically obtained during interventional procedures and are more appropriate for high-risk or symptomatic populations (123, 137). However, many of these fluids are routinely collected during diagnostic or therapeutic procedures, so their use for biomarker testing adds no extra invasiveness or risk. For certain local fluids, processing and extraction are less standardized than for plasma, compromising reproducibility and hindering translation.

Plasma was the most frequently used biofluid across both single-cancer and pan-cancer studies, with all pan-cancer studies in our survey used blood as the primary biofluid. Our summary of the literature (**Figures 2**, **3**) reveals that the single-cancer data also reveal a highly cancer-dependent pattern of biofluid selection. For urological tumors, urine was the predominant biofluid in bladder cancer studies included in our survey, with all six studies relying on this sample type, and urine usage frequency in prostate cancer studies markedly exceeded that of plasma or serum. In colorectal cancer, stool served as a major sample type alongside plasma. For cervical cancer, cervical exfoliated cells were the predominant specimen type, adopted by five studies. In addition, a subset of studies explored anatomically proximal biofluids such as breast milk for breast cancer, and bronchoalveolar lavage fluid and sputum for lung cancer. Although the number of such studies remains limited, their inclusion in our survey illustrates the diversity of sample types being investigated beyond conventional blood-based approaches.

Despite these observable trends, it must be emphasized that the substantial heterogeneity across the included studies precludes a straightforward, head-to-head comparison of diagnostic performance among different biofluids for the same cancer type. This heterogeneity manifests across multiple dimensions: validation cohort sizes vary widely (ranging from as few as 6 to nearly 9,000 participants); evidence levels differ markedly, from case-control to prospective, as color-coded in **Figure 2**; biomarker types are diverse (methylation, fragmentomics, RNA, proteins, EVs, metabolites, epidemiology and their combinations); and the stage distribution of enrolled cancer cases varies considerably across studies. In addition, control group designs are inconsistent across studies; we have broadly classified them into five categories (healthy individuals, high-risk populations, benign lesions, precancerous lesions, and other cancers) to facilitate cross-study comparison. These differences collectively make it difficult, at present, to reliably rank the real-world performance of different biofluids within a given cancer type. Nevertheless, **Figure 2** suggests a pattern in which distinct cancers show a noticeable preference for certain biofluids, likely reflecting differences in tumor biology, local shedding dynamics, and anatomical barriers. Given the heterogeneity observed, future prospective studies with head-to-head comparisons of matched biofluid panels are needed to validate these preliminary observations and to inform evidence-based biofluid selection for clinical implementation.

In summary, plasma is currently the most widely used biofluid for multi-cancer detection, whereas local body fluids may offer improved organ-specific accuracy in certain settings due to higher target enrichment and lower background noise. The choice of biofluid should therefore be guided by the target cancer type and the trade-off between detection sensitivity and sampling practicality.

## 4 Biomarker characteristics — types and characteristics of liquid biopsy biomarkers

While biofluid selection determines the source of tumor signals, identifying optimal molecular targets remains the subsequent clinical challenge. Liquid biopsy leverages a diverse array of circulating biomarkers (e.g., cfDNA, cfRNA, proteins) to enable cancer detection, with each class offering distinct advantages and limitations that provide complementary information about tumor presence, origin, and biology.

### 4.1 Overview of circulating biomarker types and their characteristics

Circulating biomarkers originate via apoptosis, necrosis, active secretion, and mechanical shedding. cfDNA can be interrogated at three levels: somatic mutations provide direct evidence of driver alterations but suffer from low abundance at early stages and poor tissue specificity (18, 138, 139); methylation patterns offer stable, tissue-specific epigenetic signatures that arise earlier in carcinogenesis and are well-suited for MCED (140, 141); and fragmentomics captures chromatin remodeling and altered nuclease activity without chemical conversion, enabling cost-effective low-pass whole-genome sequencing (lpWGS) (142). Beyond cfDNA, each additional biomarker class extends coverage into a biological domain that nucleic acid-based assays alone cannot fully address. Protein biomarkers benefit from mature, low-cost platforms but lack early-stage sensitivity and tissue specificity (16, 117). CfRNA carries rich subtype information yet is prone to degradation and lacks standardized protocols (143). CTCs allow direct characterization of intact tumor cells but are present at extremely low numbers in early disease, limiting their current utility to late-stage prognostic monitoring (144–146). EVs theoretically offer higher circulating abundance than cfDNA through their lipid-membrane protection, but non-standardized isolation and heavy background from normal cells remain major bottlenecks (147, 148). Microbiome DNA, which reflects gut or tissue-resident microbial dysbiosis associated with carcinogenesis, has recently emerged as a novel analyte, though its application remains limited (97). Metabolites represent downstream functional readouts of cellular activity and can be measured using mass spectrometry-based platforms, but they are more susceptible to pre-analytical variability and systemic physiological fluctuations (149, 150). Epidemiological variables (e.g., age, sex, lifestyle factors) are not molecular biomarkers per se but have been incorporated into multi-factorial risk models to complement molecular signals (54). Together, these biomarkers cover distinct molecular layers of tumor biology, with their strengths and limitations summarized in **Table 1**.

**Table 1:** Characteristics, analytical principles, advantages, and limitations of core liquid biopsy biomarkers.

Biomarker type	Core detection content / principle	Advantages	Limitations
cfDNA Methylation	CpG island methylation patterns, detected via bisulfite conversion, targeted sequencing, or enrichment methods.	• Strong tissue specificity, enabling precise localization of the TOO. • Stable signals; changes occur earlier than genetic mutations in early carcinogenesis. • Suitable for MCED and tissue tracing.	• Complex detection workflow (e.g., bisulfite conversion). • High cost of whole-genome methylation sequencing. • High-depth sequencing required for low-abundance samples.
cfDNA Fragmentomics	Fragment size distribution, end motifs, nucleosome footprints, preferred end coordinates, etc.	• No chemical conversion required; simple sample processing. • Can simultaneously infer epigenetic information such as methylation and histone modifications. • Suitable for lpWGS.	• Sensitive to sample processing workflows (e.g., plasma separation and storage). • High complexity in data analysis, requiring specialized algorithms (e.g., DELFI).
cfDNA Mutations	SNVs, InDels, SVs, etc. (e.g., *EGFR*, *BRAF*, *KRAS*).	• Can directly identify driver gene mutations. • Useful for targeted therapy matching and resistance monitoring.	• Weak signals in early carcinogenesis, leading to insufficient sensitivity. • Mutation profiles lack tissue specificity, making it difficult to localize the organ of origin.
cfRNA	mRNA, miRNA, snRNA, tsRNA, repetitive RNA sequences, etc.	• Multiple subtypes with high information content. • Good stability of certain RNAs (e.g., miRNA).	• RNA molecules are prone to degradation with a short half-life. • Lack of standardized extraction and detection protocols. • Low abundance in plasma.
Protein Biomarkers	CA125, CEA, CA19-9, AFP, etc., often combined in multi-marker models.	• Low detection cost, suitable for large-scale screening. • Mature platforms (e.g., ELISA, CLIA). • Can be combined with ctDNA or imaging to improve diagnostic accuracy (e.g., OncoSeek).	• Insufficient sensitivity for early-stage cancer (especially Stage I). • Poor specificity; easily interfered by benign lesions. • Weak tissue localization capability.
CTCs	Enriched via immunomagnetic beads, filtration, or density gradient centrifugation; identified by immunofluorescence or FISH.	• Allows direct analysis of intact tumor cells. • Provides tumor biological information. • Shows potential for early detection in certain cancers (e.g., lung, liver, breast).	• Extremely low numbers in early-stage cancer, making capture difficult. • High heterogeneity, prone to missed detection. • Currently mainly used for advanced-stage prognostic evaluation.
EVs	Exosomes, microvesicles, etc., containing multi-omics information like proteins, RNA, and DNA.	• High stability due to lipid membrane protection. • Carries multi-omics information, suitable for multimodal analysis. • Total circulating abundance higher than cfDNA, theoretically improving early signal capture.	• Lack of standardization in isolation and detection. • Significant interference from normal cell-derived EVs. • Most studies are retrospective, lacking prospective validation.

Data from **Figures 2**, **3** reveals how these biomarkers are actually being deployed across different cancer types, though the small number of studies per category warrants caution in interpretation. Methylation markers are the most broadly adopted class, present in nearly every cancer type surveyed. Fragmentomics are also used across nearly all cancer types surveyed, although they appear more frequently in lung cancer studies, where 5 of the 7 studies rely primarily on this approach, sometimes with methylation added as a complement. They also appear in a smaller subset of liver, gastric, colorectal, and other cancers. RNA markers are used across multiple cancer types and are often detected within EVs, which may enhance signal stability by providing physical protection against degradation. Protein markers, despite their long history in clinical diagnostics, are rarely used alone, while the majority of protein-inclusive studies integrated proteins with methylation, RNA, or epidemiological variables. EVs-associated biomarkers appear across prostate, colorectal, gastric, and breast cancers, typically in combination with RNA or protein markers, reflecting nascent but growing interest. Metabolites and microbiome DNA markers appear less frequently reported and are generally used alongside other biomarker types rather than as independent analytes. Epidemiological variables are also occasionally incorporated into risk models, usually layered on top of protein or molecular signals to improve risk stratification.

In contrast to this diversity, the pan-cancer studies in our survey show a much narrower landscape, the vast majority rely on methylation or fragmentomics, suggesting a field-wide convergence driven by the practical advantages of these cfDNA-based approaches for multi-cancer detection. However, single-cancer data remain too sparse, often with only one or two studies per cancer-type-biomarker combination and are too heterogeneous in design to support any generalized conclusion about biomarker superiority. Moreover, head-to-head comparisons of different biomarker classes for the same cancer type and the same clinical use case are virtually absent from the literature, leaving the field without a clear evidentiary basis for recommending one biomarker class over another for any given cancer.

### 4.2 Multi-omics integration in early cancer detection

Across the studies surveyed in **Figures 2**, **3**, multi-omics approaches are more commonly employed than single-biomarker strategies. Multi-omics integration of different biomarker types is a key direction in the field. Effective multi-omics integration relies on contributing distinct biological information rather than maximizing biomarker numbers. In contrast, stacking biomarkers tends to introduce redundant signals and erode the specificity of individually well-performing tests.

Biomarker complementarity based on distinct biological mechanisms enhances performance beyond single analytes. For example, a breast cancer study using SPOT-MAS integrated methylomics and fragmentomics via stacking ensemble (72). Fragmentomics reflects chromatin dysregulation, while methylation informs tissue-of-origin. In independent testing of early-stage breast cancer, this approach achieved AUC 0.91 (sensitivity 65%, specificity 96%), outperforming single-omic methods limited by low ctDNA. Another example is the GAMAD model for HCC, which integrated HepaAiQ with AFP, DCP, age, and sex and raised AUC from 0.862 to 0.952 with 86.5% sensitivity in BCLC 0/A disease, outperforming the serum-only GALAD model (63). This complementarity arises because abundant serum proteins offset low ctDNA shedding in early lesions, whereas methylation supplies the tumor-origin specificity missing from protein markers. These examples illustrate effective integration strategies built on non-redundant biological rationales. Noticeably, although these examples illustrate effective integration strategies built on non-redundant biological rationales, both are limited by retrospective or single-cohort designs and require independent external validation before clinical translation.

However, biomarker integration entails trade-offs between sensitivity gains and specificity losses. A large prospective colorectal cancer screening study enrolled nearly 10,000 average-risk participants and compared a single-analyte fecal immunochemical test (FIT), which detects hemoglobin alone, against a multitarget stool DNA test that layers *KRAS* mutations and two methylation markers onto the same hemoglobin assay (151). The multitarget test achieved significantly higher sensitivity for detecting colorectal cancer (92.3% vs. 73.8%) and advanced precancerous lesions (42.4% vs. 23.8%), but at a cost, as its specificity was 86.6%, compared with 94.9% for FIT alone. In a population-scale extrapolation to 10,000 average-risk individuals, this difference translates into roughly 760 additional false positives with the multitarget test relative to FIT, alongside the undeniable clinical benefit of detecting more potentially life-threatening lesions. Consequently, multi-omics integration involves a clinical trade-off, requiring careful balancing between sensitivity gains and specificity losses.

### 4.3 Summary of biomarker strategies

In conclusion, no single biomarker works best for every cancer type. Each has its own strengths and weaknesses. Multi-omics can boost performance if the added markers bring new biological information, but incorporating diverse markers frequently entails a statistical trade-off, where cumulative background noise can inadvertently compromise assay specificity. Single biomarkers remain useful because they are simpler and cheaper. The right choice depends entirely on the specific clinical need.

## 5 Model construction — from biomarker identification to model construction

In our previous sections, we outlined the diverse biofluids and biological characteristics of available liquid biopsy biomarkers, translating these molecules into a viable diagnostic test requires shifting our focus from *what* to measure to *how* to extract clinically meaningful signals. Building a robust diagnostic model fundamentally consists of two steps: first, screening and identifying specific targets from the chosen biomarker class, and second, optimizing algorithms to construct a model that generalizes beyond the training data. These two tasks are deeply linked. One is setting the right biological and experimental conditions to find genuine signals, and the other is selecting modeling strategies that avoid overfitting.

### 5.1 Target identification and control group design: defining the signal against clinically relevant noise

The strategy for screening and selecting specific screen targets varies fundamentally depending on the type of biomarker being utilized. For genomic biomarkers, such as cfDNA mutations, target selection typically involves identifying established driver gene alterations or designing broad panels to capture highly recurrent somatic mutations. Conversely, for epigenetic markers like cfDNA methylation, researchers employ genome-wide profiling to discover differentially methylated regions (DMRs) that are highly specific to tumor tissues compared to normal blood cells. Fragmentomics relies on mapping genome-wide cleavage patterns and size distributions to identify cancer-specific structural signatures, whereas protein-based target selection often focuses on quantifying known tumor-associated antigens against physiological baselines. However, screening for these specific targets requires a carefully designed reference background to identify a genuine tumor signal. The choice of control group determines the background noise a marker must overcome, and it should be dictated entirely by the intended clinical use of the product. It is an indispensable part of target selection, as there is no universal template and the appropriate control differs fundamentally between pan-cancer population screening and single-cancer clinical triage.

Pan-cancer screenings target asymptomatic individuals in whom cancer prevalence is relatively low (139). In this setting, any false positive triggers costly downstream workups and psychological burden, making specificity the primary design constraint. Circulating Cell-free Genome Atlas (CCGA) addressed this by building a control cohort representative of real-world complexity, with cancer-free participants from its own enrolment supplemented by women undergoing routine mammography in the STRIVE study, capturing the diversity of ages, hormonal statuses, and benign comorbidities present in an actual screening population (139). This breadth of non-cancer representation allowed the final classifier to maintain high specificity against complex physiological backgrounds.

Single-cancer studies operate in distinct clinical contexts, where participants present specific indications. Participants typically present with a specific indication, such as a pulmonary nodule, hematuria, or elevated PSA, and the clinical question shifts from “could this person have cancer?” to “should this person undergo invasive biopsy?” Control design must therefore be stratified around the precise confounders that create clinical ambiguity. Lung cancer studies need to include heavy smokers with Chronic Obstructive Pulmonary Disease (COPD) or inflammatory granulomas to test whether the model can separate malignant nodules from benign inflammatory lesions (26, 152). Bladder cancer studies must include patients with hematuria from stones or urinary tract infections to prevent bleeding signals from being misread as tumor shedding (153, 154). In prostate cancer, where overdiagnosis is a recognized harm, some deliberately assign low-grade indolent cancers (Gleason 3 + 3) to the control group, training the classifier to distinguish clinically aggressive disease from lesions that would not benefit from immediate intervention (43). Beyond benign-malignant differentiation, single-cancer studies often incorporate longitudinal samples spanning precancerous states, including advanced adenomas in colorectal cancer (82, 155, 156), atrophic gastritis or intestinal metaplasia in gastric cancer (44, 49), and cervical intraepithelial neoplasia grades in cervical cancer (157), to test whether model scores correlate stepwise with malignant progression risk.

Integrating rigorous controls into target discovery ensures markers indicate malignancy rather than inflammation or aging artifacts. Pan-cancer and single-cancer designs are therefore complementary, the former establishes a broad specificity baseline across diverse comorbidity distributions, the latter maps the specific confounders that any pan-cancer model must resolve at the organ level. This rigorous target identification lays a clean and reliable data foundation for the subsequent step of algorithmic modeling.

### 5.2 Feature selection and algorithm matching

High-throughput platforms such as WGS and cfMeDIP-seq can generate millions of candidates features from a single sample (158). This abundance creates a dimensionality trap where excessive features fail to increase discriminability and lead to overfitting (159, 160). Consequently, feature dimensionality reduction is essential, categorized into biological and statistical filtering. Biological filtering relies on prior mechanistic knowledge to pre-select functionally relevant targets, whereas statistical filtering utilizes mathematical algorithms to eliminate redundant or low-contributing variables from whole data. The empirical principle that simpler, strategically reduced feature sets generalize better than exhaustive high-dimensional ones, Occam’s Razor in machine learning, is well supported. In the 2012 Sage Bionetworks/DREAM breast cancer prognosis challenge, models relying on a small number of core gene modules outperformed those using whole-genome expression profiles; the latter achieved excellent training fit but collapsed in independent validation due to overfitting (161, 162).

Methodical feature reduction is equally critical in early cancer detection. However, it is important to clarify that “simpler” does not strictly mean reducing the test to a single-digit number of markers; rather, it means a deliberate distillation of whole-omics data into highly informative, targeted subsets using either or both of these two aforementioned strategies. For instance, in CCGA2 study (139), which heavily utilized a statistical filtering approach, researchers recognized the dimensional trap of WGBS, which spans roughly 30 million CpG sites across the genome. Feeding the entire genome into a classifier would result in high background noise and shallow sequencing depth. To overcome this, they filtered the genome down to a targeted panel of approximately 1 million highly informative CpGs across ~100,000 distinct regions by computationally identifying the most discriminative methylation signals across a vast dataset. While 1 million CpGs is still a massive feature set, this deliberate reduction from the whole genome allowed for deeper sequencing and stable machine learning classification across more than 50 cancer types without overfitting. Conversely, when assay designs prioritize targeting specific known mechanisms, they may lean heavily on biological filtering. For targeted single-cancer screening, such as stool-based colorectal cancer tests, the optimal feature set can be constrained by biological rationale to a handful of features, such as hemoglobin, a *KRAS* mutation, and two specific methylation markers with established mechanistic relevance in early tumorigenesis (151). In both scenarios, the feature set is actively filtered based on statistical optimization, biological rationale, or a synergistic combination of both, rather than simply feeding all available raw data into an algorithm.

Feature selection and algorithm choice are jointly constrained decisions. A widely used rule of thumb is that the number of training data points should be 10 times the number of weights (163), but some authors have suggested a more conservative minimum ratio of 50 (164). Increased algorithmic complexity does improve performance when sample sizes and feature dimensionality are sufficient, but this advantage disappears under data-limited conditions (165, 166). When predictive variables number fewer than ten to twelve, complex models such as SVMs, neural networks, and random forests exhibit significant overfitting even with over 200 positive events; simple logistic regression achieves stable estimates with as few as 20 to 50 events (160). The choice of algorithm complexity should consider both the sophistication of the model and the true information content of the data.

A major pitfall is data leakage, where test set information is inadvertently incorporated into training. This occurs when feature selection or model tuning is performed across the entire dataset before cross-validation, allowing the model to “see” the answers it is meant to predict. The canonical cautionary example is a 2002 *Lancet* study reporting near-perfect ovarian cancer detection using serum proteomic patterns (167–169). Subsequent reanalysis demonstrated that the model’s reported success was not driven by tumor biology, but by the model’s reliance on systematic batch effects and measurement noise that differed between training and validation samples. The assay’s clinical validity was ultimately overturned when it failed to replicate on truly independent data. To prevent such failures, strict compartmentalization of all feature selection and optimization steps within the training fold, coupled with fully blind independent validation, are not optional safeguards. They are foundational requirements that determine real-world product success far more decisively than the choice of algorithm itself.

## 6 Clinical validation — clinical trials and translation status

Liquid biopsy products must undergo progressively stringent clinical validation before clinical implementation.

### 6.1 Hierarchy of clinical trial evidence: the ladder from technical signals to clinical application

Clinical validation of liquid biopsy tests follows three levels of evidence. The first level is the case-control study which uses samples from individuals with known disease status to provide an initial assessment of test performance under rigorously controlled conditions. The second level is the prospective real-world population cohort study. These studies evaluate the test in actual target screening populations to observe its true performance and feasibility without artificially setting disease prevalence. The third level is the randomized controlled trial (RCT) which serves as the gold standard to assess whether the screening ultimately provides tangible clinical benefit such as reducing the incidence of late-stage cancers.

GRAIL’s clinical development program illustrates this evidence hierarchy. The company initially conducted the CCGA3 study as a first level case-control validation to establish the baseline accuracy of their test (114). They subsequently advanced to the second level with the PATHFINDER 1/2 studies to evaluate real-world screening performance in tens of thousands of asymptomatic adults (115, 116). Concurrently they launched the NHS-Galleri trial as a massive third level RCT to definitively determine if the blood test can truly reduce the burden of late-stage cancer in the general population (170).

### 6.2 The inevitable performance degradation from case-control to real-world settings

The transition from case-control studies to real-world screening involves substantial performance degradation. When liquid biopsy technologies move from level I case-control studies to level II real-world screening in asymptomatic populations, performance degradation is almost inevitable, with the most pronounced drop occurring in sensitivity. For instance, GRAIL’s CCGA3 study reported an overall sensitivity of 51.5% in a case-control setting, but this fell to approximately 29.0% (35/121) in the prospective real-world PATHFINDER cohort (114, 115). Similarly, the MERCURY-based CanScan achieved a sensitivity of 87.4% in its validation set, yet this declined to 53.5% in the real-world prospective JinLing cohort (111, 112). These figures clearly illustrate that sensitivity is substantially diluted in low-prevalence screening scenarios.

Even clearing this hurdle does not guarantee clinical benefit. Even if a test can detect early-stage cancers in asymptomatic real-world populations and maintain an acceptable PPV, this does not automatically translate into clinical benefit. It must be further validated through a RCT to determine whether it ultimately reduces cancer mortality. The South Korean ultrasound screening program for thyroid cancer serves as a sobering lesson. It led to a 15-fold increase in thyroid cancer incidence without any corresponding decline in mortality (171–173). This indicates that early detection does not inherently reduce mortality. Without confirmation from RCT, widespread adoption may instead exacerbate the risks of overdiagnosis and overtreatment.

### 6.3 Current landscape of clinical evidence across cancer types

Nowadays, across the major cancer types for which liquid biopsy early detection has been pursued, the clinical evidence landscape follows a broadly consistent pattern. Case-control studies are abundant. Prospective real-world cohort data are comparatively sparse. At present, there is no RCT evidence for any single-cancer liquid biopsy test. The only large-scale RCT in this field, NHS-Galleri, targets multi-cancer detection and has not yet reported its primary endpoint (170). This evidence ceiling does not reflect scientific inadequacy but rather structural necessity, because cancer screening RCTs require enrolment in the hundreds of thousands, follow-up lasting years to decades, and mortality endpoints that cannot be accelerated.

However, even with this common constraint, cancer types have progressed to very different degrees. Colorectal cancer screening shows the highest clinical maturity. The ECLIPSE trial compared a new blood test against colonoscopy in over 20,000 participants and met the FDA’s prespecified performance criteria (58), Shield was subsequently approved by the FDA in 2024. Similarly, the CRC PREVENT trial validated stool RNA test ColoSense across nearly 9,000 average risk individuals (56). Liver cancer research benefits from clearly defined high risk groups like patients with chronic hepatitis and cirrhosis. This allows for massive prospective studies such as the landmark aMAP-2 trial which tracked over 13,000 participants longitudinally to deliver highly reliable real-world data (174). Lung cancer research effectively utilizes high risk smoking cohorts already in low dose CT screening programs. Studies like the DELFI assay have evaluated test performance under actual screening conditions providing a strong foundation for using blood tests alongside traditional imaging (175). For gastric cancer, the situation is distinct. A pragmatic randomized trial in ~240,000 adults in Taiwan found that *Helicobacter pylori* stool-antigen screening did not significantly reduce gastric-cancer incidence or mortality (RR 0.86; 95% CI 0.68–1.10) (176). For blood-based liquid biopsy, the GUIDE study has validated the GutSeer assay in a prospective cohort of 846 participants (119). For stool-based assays, prospective studies are ongoing, with preliminary data showing encouraging sensitivity (e.g.: NCT06943807).

Not all cancer types have advanced at the same pace. Breast and prostate cancers face distinct structural barriers that have slowed their progression toward second-tier evidence. For breast cancer, the absence of an organ-proximal biofluid means that circulating signals from early lesions are heavily diluted in blood, with sensitivity for Stage I disease remaining consistently limited across current assays (114, 177); beyond this biological constraint, mammography has established a strong performance benchmark that any liquid biopsy approach must demonstrably exceed (178). Prostate cancer presents a unique landscape because the widespread use of PSA testing has shifted liquid biopsy away from primary screening (179). These tests now primarily offer risk stratification and biopsy support for men with intermediate PSA levels. Urine based products like SelectMDx and ExoDx have accumulated prospective data from thousands of participants in this specific triage role (42, 43). Despite this progress, researchers have not yet established prospective evidence to support liquid biopsy as a first line screening tool for the asymptomatic general population.

## 7 Discussion

Our review provides a data-driven synthesis of approximately 80 single-cancer and 20 pan-cancer studies. We show that plasma dominates multi-cancer detection, while local fluids offer advantages for organ-specific settings, though head-to-head comparisons are absent. Methylation and fragmentomics are the standards for pan-cancer tests, whereas single-cancer studies explore a wider range including proteins, RNA, and EVs. Methodological heterogeneity across studies, including cohort size, control design, and evidence level, severely limits cross-study comparability. Prospective real-world validation remains rare, and randomized controlled trial evidence is particularly scarce. Along the translational pathway, our review identifies four interconnected decision points that collectively determine the success of a liquid biopsy assay: biofluid selection, biomarker characteristics, model construction, and clinical validation, each of these stages entails its own biological and methodological challenges. Yet biological and methodological considerations, the successful translation of liquid biopsy into routine cancer screening hinges on addressing four interconnected translational bottlenecks: economic viability, analytical standardization, regulatory harmonization, and health-system readiness. These factors are often treated as downstream implementation details, but they are in fact integral to the translational pathway and must be addressed in parallel with scientific development.

Health economics and reimbursement represent the primary gatekeeper. While cost-effectiveness models, such as the US-based analysis of the Galleri test with an Incremental Cost-Effectiveness Ratio (ICER) of approximately $66,000 per Quality-Adjusted Life Year (QALY), provide useful benchmarks, they often overlook the total budget impact and upfront infrastructure investment required for population-wide deployment (180). The economic tension is also present in China, where affordability varies drastically between urban eastern and rural western regions (181). A strategy deemed cost-effective in one setting may be financially prohibitive in another, underscoring the urgent need to embed prespecified economic endpoints, covering both ICER and total budgetary outlays, into prospective screening trials to inform tiered reimbursement policies.

Assay standardization and laboratory quality control pose another critical challenge. The rapid proliferation of diverse biomarkers, including methylation, fragmentomics, proteins, and EVs, coupled with continuous platform innovation, has outpaced the development of unified reference materials and external proficiency testing protocols. Without standardized pre-analytical workflows and harmonized quality metrics, cross-study comparability remains severely compromised. This lack of standardization directly undermines the reproducibility of clinical validation and complicates the aggregation of evidence for regulatory review.

Regulatory pathways for multi-cancer early detection tests remain fragmented globally. Inconsistencies in evidentiary requirements, particularly regarding the management of screen-positive but imaging-negative individuals, create substantial hurdles for both payer coverage decisions and multinational clinical development. While large-scale pan-cancer trials have recently emerged in China (e.g., Jinling and FuSion projects) to generate local evidence (112, 120), the absence of harmonized validation standards across jurisdictions (e.g., NMPA vs. FDA vs. CE marking) impedes the global translation of promising technologies.

Finally, health-system implementation capacity determines whether a clinically valid test translates into population-level benefit. Implementation entails hidden costs rarely captured in economic models, including logistics for sample collection and transport, provider training, and robust patient navigation systems to manage diagnostic workup after positive results. In China, a large real-world program reported only ~22% adherence to follow-up colonoscopy after positive stool-based screening, starkly illustrating how implementation gaps can nullify the theoretical benefits of even cost-effective tests (182). Improving this adherence requires dedicated infrastructure and decision-support tools, costs that must be factored into comprehensive evaluation frameworks.

Future research should prioritize direct comparisons of biofluids, biomarker classes, and integration strategies within identical cohorts. To bridge the population generalizability gap, large-scale prospective screening must be implemented across diverse geographic settings, incorporating prespecified economic endpoints, including cost-effectiveness and budget impact, alongside clinical performance metrics to inform both regulatory approval and reimbursement decisions. Ultimately, successful translation hinges on coordinated efforts among developers, regulators, payers, and providers, extending beyond the laboratory into health policy, health economics, and health system strengthening to close the gap between innovation and real-world clinical utility.
